# Supplementary material for: Effect of Different Anchorage Reinforcement Methods on Long-Term Maxillary Whole Arch Distalization with Clear Aligner: A 4D Finite Element Study with Staging Simulation
Source: Bioengineering (Basel). 2023 Dec 20;11(1):3. doi: 10.3390/bioengineering11010003 (PMC10813679; doi:10.3390/bioengineering11010003)
Supplement: Supplementary file 1 [file bioengineering-11-00003-s001.zip › Table S1.pdf]

Table S1. Three-dimensional displacement (mm) of the crown points and rotation (°) of the long axis of each tooth during the total arch distalization of the 4 groups. Tooth numbering according to the FDI tooth numbering system. The positive axis was determined by the right-handed rule.

Group	Tooth	Direction	Steps	Displacement (mm)							Rotation (°)						
				10	20	30	40	50	60	70	10	20	30	40	50	60	70
	1	X		-0.03	-0.07	-0.11	-0.16	-0.38	-0.19	-0.60	1.21	4.05	7.53	9.38	10.16	6.00	0.65
		Y		-0.24	-0.82	-1.51	-1.80	-2.06	-1.32	0.87	-0.01	-0.11	-0.37	-0.36	0.68	0.09	2.40
		Z		-0.18	-0.66	-1.31	-1.84	-2.00	-1.42	-1.08	-0.06	-0.17	-0.26	-0.35	-0.72	-0.32	-0.90
	2	X		-0.02	-0.07	-0.10	-0.34	-0.50	-0.38	-1.23	1.03	3.63	6.70	7.50	8.48	5.01	-0.97
		Y		-0.23	-0.76	-1.38	-1.61	-1.85	-1.24	1.03	-0.01	-0.06	-0.24	0.88	1.68	1.24	4.01
		Z		-0.15	-0.53	-1.04	-1.23	-1.24	-0.80	-0.42	-0.07	-0.23	-0.37	-0.83	-1.18	-0.82	-1.55
	3	X		-0.06	-0.21	-0.34	-0.68	-0.68	-0.53	-0.49	1.20	4.03	6.37	5.66	3.03	0.33	0.33
		Y		-0.20	-0.74	-1.37	-0.98	-0.30	0.51	0.56	0.37	1.33	2.16	4.34	4.40	3.78	3.63
		Z		-0.10	-0.30	-0.49	-0.53	-0.35	-0.33	-0.37	-0.36	-1.22	-1.93	-2.89	-2.63	-1.94	-1.85
Control group	4	X		-0.07	-0.17	-0.35	0.42	0.46	0.41	0.41	1.38	3.76	5.03	-0.48	-4.64	-4.26	-4.12
		Y		-0.26	-0.81	-1.28	-0.37	0.55	0.51	0.51	0.47	1.14	1.36	-2.14	-2.23	-1.77	-1.63
		Z		-0.06	-0.10	-0.19	-0.08	-0.18	-0.18	-0.18	-0.27	-0.55	-0.55	1.89	1.77	1.36	1.23
	5	X		-0.10	-0.19	-0.04	0.94	0.87	0.83	0.82	1.28	3.35	1.11	4.85	4.77	4.59	4.47
		Y		-0.26	-0.83	-0.01	0.72	0.72	0.71	0.70	0.59	1.14	0.26	-3.36	-2.77	-2.46	-2.31
		Z		-0.05	-0.10	-0.09	-0.20	-0.21	-0.21	-0.20	-1.06	-1.47	-0.88	11.12	8.88	7.70	7.12
	6	X		-0.12	0.05	0.18	0.90	0.87	0.85	0.85	0.82	-2.57	-0.72	-3.11	-2.68	-2.37	-2.21
		Y		-0.24	0.39	1.08	0.62	0.64	0.62	0.62	0.59	-0.28	-0.63	-2.04	-1.17	-0.65	-0.34
		Z		-0.05	-0.15	-0.40	-0.25	-0.22	-0.21	-0.21	0.61	0.25	0.51	-1.70	-0.63	-0.01	0.37
	7	X		0.16	0.15	-0.02	0.44	0.44	0.43	0.41	-5.92	-8.59	-5.92	-4.05	-4.43	-4.65	-4.78
		Y		0.97	1.55	1.13	0.66	0.69	0.71	0.73	-1.39	-1.66	-0.90	-2.41	-2.12	-1.88	-1.70

		Z	-0.37	-0.66	-0.64	-0.68	-0.70	-0.70	-0.69	0.03	0.23	0.24	-0.59	-0.45	-0.35	-0.27
Buccal temporary anchorage device group	1	X	-0.02	-0.04	-0.06	-0.07	-0.25	-0.05	-0.49	1.08	2.96	5.96	8.24	9.30	4.88	1.90
		Y	-0.12	-0.62	-1.14	-1.27	-1.64	-0.81	1.71	-0.02	-0.20	-0.47	-0.80	0.01	-0.39	1.80
		Z	-0.22	-0.73	-1.35	-1.98	-2.14	-1.46	-1.80	-0.04	-0.07	-0.13	-0.13	-0.45	-0.08	-0.60
	2	X	-0.00	-0.02	-0.03	-0.31	-0.44	-0.27	-1.15	0.94	2.88	5.38	6.67	7.74	3.50	-1.73
		Y	-0.12	-0.62	-1.01	-1.14	-1.50	-0.68	1.84	-0.17	-0.28	-0.57	0.35	0.92	0.98	3.12
		Z	-0.19	-0.59	-1.06	-1.30	-1.41	-0.94	-0.95	0.01	-0.08	-0.14	-0.56	-0.84	-0.59	-1.04
	3	X	-0.02	-0.10	-0.22	-0.47	-0.54	-0.48	-0.46	0.72	2.59	5.13	4.85	2.55	-0.39	-0.20
		Y	-0.07	-0.49	-0.86	-0.67	-0.02	1.02	1.04	-0.02	0.42	1.25	2.81	3.15	2.86	2.75
		Z	-0.12	-0.31	-0.54	-0.48	-0.38	-0.50	-0.51	-0.07	-0.54	-1.26	-1.98	-1.84	-1.21	-1.14
	4	X	-0.02	-0.09	-0.22	0.50	0.56	0.51	0.51	0.84	2.11	3.24	-1.88	-5.79	-5.15	-4.87
		Y	-0.14	-0.59	-0.98	-0.06	0.82	0.75	0.73	0.23	0.66	0.95	-2.13	-1.91	-1.30	-1.04
		Z	-0.04	-0.08	-0.13	-0.11	-0.24	-0.22	-0.22	-0.12	-0.34	-0.43	1.79	1.38	0.82	0.57
	5	X	-0.04	-0.12	0.02	0.66	0.60	0.57	0.57	0.74	1.62	-1.59	1.18	1.06	1.05	0.84
		Y	-0.14	-0.62	0.45	1.07	1.06	1.05	1.02	0.30	0.83	-0.28	-3.50	-3.15	-2.90	-2.51
		Z	-0.04	-0.07	-0.15	-0.25	-0.26	-0.28	-0.29	-0.53	-1.43	-0.27	12.84	11.22	10.10	8.29
	6	X	-0.06	0.12	0.17	0.74	0.72	0.70	0.70	0.39	-2.40	-2.02	-3.38	-3.48	-3.20	-3.05
		Y	-0.14	0.57	1.16	0.92	0.93	0.92	0.92	0.34	-0.30	-0.52	-2.09	-1.26	-0.78	-0.55
		Z	-0.04	-0.20	-0.37	-0.30	-0.26	-0.24	-0.24	0.37	0.23	0.62	-1.48	-0.50	0.06	0.33
	7	X	0.19	0.14	-0.11	0.39	0.41	0.40	0.38	-6.63	-10.25	-7.31	-6.11	-.33	-6.50	-6.64
		Y	1.09	1.87	1.42	1.11	1.12	1.13	1.14	-1.63	-1.84	-0.40	-2.20	-2.02	-1.81	-1.63
		Z	-0.42	-0.79	-0.75	-0.78	-0.80	-0.80	-0.79	0.01	0.31	0.54	-0.29	-0.22	-0.13	-0.05
Palatal temporary anchorage	1	X	-0.02	-0.06	-0.09	-0.10	-0.49	-0.49	-0.88	0.44	2.55	5.63	5.65	6.00	1.12	-6.00
		Y	-0.04	-0.48	-1.09	-1.20	-1.39	-0.50	1.88	0.04	-0.01	-0.18	-0.07	1.72	1.98	4.00
		Z	-0.05	-0.39	-0.96	-1.17	-1.21	-0.49	0.31	-0.04	-0.13	-0.22	-0.26	-0.88	-0.79	-1.40
	2	X	-0.01	-0.05	-0.10	-0.29	-0.56	-0.49	-1.42	0.42	2.38	5.01	4.31	4.80	0.01	-7.16

device		Y	-0.06	-0.46	-1.04	-0.88	-1.12	-0.22	2.26	-0.02	-0.04	0.01	0.97	2.19	2.63	5.25
group		Z	-0.05	-0.34	-0.78	-0.72	-0.66	-0.06	0.62	-0.02	-0.15	-0.35	-0.67	-1.17	-1.11	-1.94
	3	X	-0.01	-0.10	-0.23	-0.44	-0.55	-0.55	-0.50	0.39	2.38	4.75	3.11	-0.57	-4.95	-4.88
		Y	-0.05	-0.49	-1.04	-0.77	-0.02	1.16	1.23	0.04	0.53	1.54	2.91	3.66	3.63	3.38
		Z	-0.05	-0.18	-0.37	-0.11	0.27	0.38	0.30	-0.08	-0.61	-1.45	-1.98	-2.00	-1.45	-1.28
	4	X	-0.01	-0.05	-0.21	0.66	0.64	0.56	0.56	0.64	2.68	4.00	-2.20	-6.31	-5.59	-5.42
		Y	-0.09	-0.52	-1.00	0.14	1.03	0.90	0.89	0.16	0.63	0.94	-2.72	-2.49	-1.88	-1.75
		Z	-0.04	-0.07	-0.14	-0.04	-0.07	0.06	0.07	-0.08	-0.27	-0.37	2.32	1.94	1.40	1.28
	5	X	-0.02	-0.03	0.11	1.44	1.27	1.16	1.14	0.54	2.05	-1.09	2.29	2.20	1.99	1.84
		Y	-0.09	-0.53	0.37	1.14	1.14	1.11	1.09	0.22	0.52	-0.55	-6.02	-5.13	-4.64	-4.51
		Z	-0.03	-0.06	-0.11	-0.29	-0.30	-0.34	-0.34	-0.39	-0.53	0.90	23.13	19.52	17.33	16.70
	6	X	-0.03	0.20	0.32	1.24	1.21	1.18	1.17	0.23	-3.78	-2.99	-4.45	-5.16	-4.84	-4.66
		Y	-0.10	0.68	1.46	1.06	1.05	1.02	1.00	0.23	-0.72	-0.89	-5.04	-4.79	-4.52	-4.35
		Z	-0.04	-0.23	-0.48	-0.39	-0.38	-0.38	-0.36	0.27	0.03	0.62	-4.25	-4.02	-3.79	-3.64
	7	X	0.22	0.29	-0.07	0.53	0.61	0.62	0.61	-6.87	-11.52	-9.72	-8.35	-8.37	-8.39	-8.41
		Y	1.13	1.96	1.66	1.31	1.30	1.30	1.31	-1.80	-2.24	-0.81	-4.77	-5.07	-5.06	-5.01
		Z	-0.44	-0.85	-0.88	-0.89	-0.92	-0.92	-0.92	-0.03	0.29	0.63	-0.97	-1.09	-1.08	-1.06
	1	X	-0.02	-0.04	-0.08	-0.06	-0.30	-0.16	-0.54	0.77	3.12	6.26	8.50	9.27	4.68	0.50
		Y	-0.11	-0.58	-1.22	-1.54	-1.82	-0.98	1.16	0.01	-0.13	-0.34	-1.34	-0.18	-0.47	1.70
		Z	-0.12	-0.50	-1.12	-1.71	-1.87	-1.19	-1.06	-0.04	-0.11	-0.20	0.21	-0.24	0.14	0.40
Class II	2	X	-0.02	-0.05	-0.10	0.42	0.28	0.47	-0.41	0.70	2.86	5.55	6.32	7.50	3.26	-2.25
elastic		Y	-0.11	-0.56	-1.16	-1.01	-1.41	-0.63	1.79	-0.05	-0.11	-0.18	-2.80	-2.22	-2.19	0.19
group		Z	-0.10	-0.41	-0.90	-0.99	-1.08	-0.58	-0.37	-0.03	-0.15	-0.31	0.85	0.50	0.88	0.54
	3	X	-0.01	-0.07	-0.22	-0.48	-0.57	-0.49	-0.48	0.60	2.71	5.20	3.71	1.94	-0.09	0.15
		Y	-0.07	-0.47	-1.04	-0.70	-0.06	0.74	0.76	0.03	0.45	1.38	2.33	3.07	2.64	2.47
		Z	-0.07	-0.22	-0.44	-0.57	-0.52	-0.49	-0.50	-0.09	-0.60	-1.39	-2.27	-2.29	-1.71	-1.64

4	X	-0.02	-0.08	-0.23	0.35	0.38	0.29	0.28	0.82	2.85	4.11	0.07	-4.00	-3.53	-3.37
	Y	-0.13	-0.58	-1.02	-0.19	0.66	0.60	0.61	0.23	0.73	0.84	-2.88	-2.99	-2.51	-2.39
	Z	-0.05	-0.08	-0.16	-0.14	-0.28	-0.29	-0.29	-0.12	-0.33	-0.27	2.63	2.25	1.86	1.76
5	X	-0.04	-0.07	0.05	0.54	0.48	0.42	0.40	0.71	2.82	0.15	5.01	4.72	4.45	4.33
	Y	-0.13	-0.54	0.17	0.66	0.63	0.60	0.59	0.32	0.80	-0.04	-2.64	-1.90	-1.51	-1.37
	Z	-0.04	-0.06	-0.09	-0.29	-0.31	-0.30	-0.30	-0.59	-0.98	-0.46	9.52	8.17	7.59	7.38
6	X	-0.06	0.13	0.26	0.59	0.62	0.60	0.59	0.38	-3.53	-3.42	-5.71	-6.04	-5.68	-5.50
	Y	-0.14	0.61	1.34	1.02	0.96	0.94	0.93	0.34	-0.55	-0.82	-2.40	-1.33	-0.82	-0.54
	Z	-0.04	-0.21	-0.46	-0.34	-0.28	-0.27	-0.26	0.39	0.18	0.60	-1.60	-0.47	0.08	0.37
7	X	0.19	0.15	-0.08	0.31	0.31	0.30	0.27	-6.66	-11.15	-9.16	-7.12	-7.27	-7.39	-7.51
	Y	1.09	1.91	1.62	1.30	1.25	1.25	1.25	-1.65	-2.06	-0.79	-1.94	-1.73	-1.45	-1.18
	Z	-0.42	-0.81	-0.83	-0.85	-0.87	-0.88	-0.88	0.01	0.32	0.58	-0.40	-0.33	-0.24	-0.15
